# *Angelica acutiloba* Kitagawa Extract Attenuates DSS-Induced Murine Colitis

**DOI:** 10.1155/2016/9275083

**Published:** 2016-05-17

**Authors:** Jong-Chan Jang, Kang Min Lee, Seong-Gyu Ko

**Affiliations:** ^1^Department of Korean Medicine, College of Korean Medicine, Kyung Hee University, 26 Kyungheedae-ro, Dongdaemun-gu, Seoul 130-701, Republic of Korea; ^2^Laboratory of Clinical Biology and Pharmacogenomics, College of Korean Medicine, Kyung Hee University, 26 Kyungheedae-ro, Dongdaemun-gu, Seoul 130-701, Republic of Korea

## Abstract

We examined the protective effects of* Angelica acutiloba *Kitagawa (AAK) extract on a murine model of acute experimental colitis. Colitis was induced by 4% dextran sulfate sodium (DSS) in the drinking water of male C57BL/6 mice, for 7 consecutive days. Oral administration of AAK extract (500 mg/kg/day) significantly alleviated DSS-induced symptoms such as anorexia, weight loss, events of diarrhea or bloody stools, and colon shortening. Histological damage was also ameliorated, as evidenced by the architectural preservation and suppression of inflammatory cell infiltration in colonic samples. Treatment improved the colonic mRNA expression of different inflammatory markers: cytokines, inducible enzymes, matrix metalloproteinases, and tight junction-related proteins. In the isolated serum, IgE levels were downregulated. Collectively, these findings indicate the therapeutic potentials of AAK as an effective complementary or alternative modality for the treatment of ulcerative colitis.

## 1. Introduction

Ulcerative colitis (UC) is an idiopathic disorder that involves a proximal extension of chronic inflammation in the colonic mucosa. Major manifestations of UC include bloody diarrhea, rectal bleeding, frequent stools, tenesmus, and pain in the lower abdomen, all of which undergo repeated relapses following exacerbation and remission of the disorder [1, 2]. Although the exact etiology of UC is yet to be fully understood, the general consensus is that a combination of the following factors leads to its development: genetic risks of maternal inheritance, environmental variability, and dysbiosis [3, 4]. Treatment of the disease mainly consists of conventional pharmaceutical approaches, as well as surgical interventions for those who do not respond to the initial therapeutics. For UC patients exhibit an impaired quality of life, treatment is mostly aimed at restoring it, through induction and maintenance of the remission phase. Drugs currently in use for patients with UC can be categorized as anti-inflammatory drugs, immunosuppressants, pro/antibiotics, and biologic agents [5]. However, in most cases, the underlying mechanisms are still not clear, and long-term efficacy is achieved in only approximately one-third of the patients [4]. Current options are also hindered by undesired adverse effects such as metabolic changes, increased risk of infections or lymphomas, or even higher mortality [6]. Therefore, it is imperative that effective complementary and alternative measures be developed to remedy such defects.

Animal models of UC have proven to be useful in terms of understanding the pathophysiology of UC, not to mention testing the* in vivo* potency of possible therapeutic agents. While murine models are most frequently used, a UC-like phenotype is induced via chemical administration or bacterial infection [4, 7]. Dextran sulfate sodium (DSS), trinitrobenzene sulfonic acid (TNBS), oxazolone, and acetic acid are major colitogenic substances that cause injury to the intestinal epithelium [8]. The most widely used is the DSS, since it is relatively simple in regard to administration (usually through drinking water), besides control of dosage and duration. Rather than directly causing luminal inflammation* per se*, DSS exposes the lamina propria and submucosa to enteric antigens and bacteria by breaking down the epithelial barrier. Many of the consequent inflammatory responses resemble those of the human UC, as evidenced by exhibition of characteristic features such as weight loss, diarrhea, fecal occult blood, and intestinal shortening [9, 10]. Histopathologic features of DSS-induced colitis include mucin depletion, epithelial degeneration, and neutrophil infiltration [10]. The pathogenesis of DSS-induced colitis involves defective alterations in the tight junction complex and marked increases in the expression of inflammatory cytokines [10, 11]. With such keen resemblance to the human-derived UC, the preclinical model of DSS-induced colitis has been validated as relevant for the translation of murine data into human disease [1, 7] and thus has been adopted in this study.

The use of complementary and alternative medicine (CAM) in patients with inflammatory bowel disease (IBD) is quite common, with a rate over 40% in North America [12]. Herbal therapy is the single most used modality of CAM, followed by homeopathy, naturopathy, and dietary supplements [13, 14]. The primary reason behind such unconventional medicine use has been reported to be direct disease-related benefits, in addition to psychological factors such as the need for greater autonomy or emphasis on holistic approaches [14, 15]. Unpurified herbal decoctions contain biologically active compounds that may display antioxidative, anti-inflammatory, and prebiotic properties [13, 16]. In this study, we have tested the effects of oral* Angelica acutiloba *Kitagawa (AAK) extract on a DSS-induced colitis model. The Japanese AAK belongs to the same genus as the Korean* A. gigas* and the Chinese* A. sinensis*, all of which have been substituted by one another for* Angelicae radix *(*Angelica* root) [17]. While* A. gigas* is well-known for its active ingredient decursin and decursinol angelate, AAK is differentiated by a relatively low content of both, and a relatively high content of essential oils, organic acids, and polyphenols like* z*-ligustilide,* n*-butylidenephthalide, ferulic acid, and chlorogenic acid [18]. Previously reported bioactivities of the AAK extract include antitumor [19], anticomplementary [20], immunostimulatory [21], antidiabetic [22], antiobesity [23], and, most importantly, a considerable anti-inflammatory activity [17, 24–26]. Here we demonstrate for the first time that orally administered AAK extract significantly suppresses intestinal inflammation in the DSS-induced acute colitis model. Our findings may represent a novel efficacious and reasonable confrontation for the standard treatment of UC, as its cost of nonadherence and opportunity loss is substantial, and still on the rise [27].

## 2. Material and Methods

### 2.1. Experimental Animals

Male, 6-week-old C57BL/6 mice weighing at an average of 23 g (±1 g) were obtained from Central Lab Animal Inc. (Seoul, Korea). Mice were housed in a pathogen-free facility with a light/dark cycle of 12 h, under ambient temperature (21–23°C) and humidity (55–60%). All animals had access to food and water* ad libitum* and were acclimated for 2 weeks before the experiment.

### 2.2. Sample Preparation

Dried root of AAK (500 g) was obtained from Kyung Hee Herb Pharm (Seoul, Korea). The root was then cut down into a proper size and extracted as follows. Briefly, the sections were immersed in 30% EtOH and extracted 2 times for 2 h each, using a reflux extractor (GLHMP-F1000, Global Lab, Siheung, Korea). The extract was further concentrated with an evaporator (Rotavapor® R-220, BÜCHI Labortechnik AG, Flawil, Switzerland), followed by a filtration step and subsequent freeze-drying (LP30, Ilshin Biobase Co., Yangju, Korea) at 5 mm Torr (yield = 21.3%). For chromatographic separation, 100 mg of freeze-dried sample powder was dissolved in 1 mL MeOH, sonicated for 30 min, and filtered through a PVDF membrane.

### 2.3. High Performance Liquid Chromatography (HPLC)

**Figure 1:**
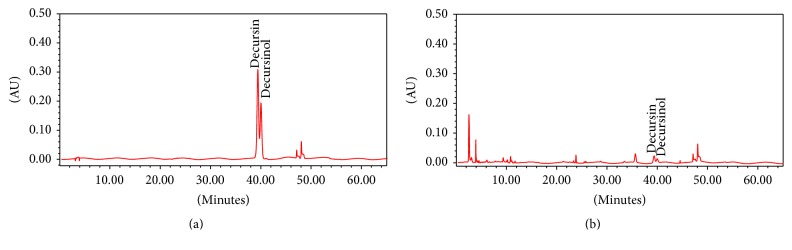
HPLC-PDA profiles of the index compound decursin, decursinol angelate, and the ethanol extract of AAK. Chromatograms of (a) decursin, decursinol angelate, and (b) AAK were monitored at 330 nm. Quantitative content of decursin and decursinol angelate in AAK was <0.001%.

Chromatographic separation of the extract was performed using a separations module (2690, Waters, MA, USA) and 5 *μ*m column (Nucleosil® 100-5 C18, MACHEREY-NAGEL, Düren, Germany). Using solvents A (water) and B (acetonitrile), the gradient program was run as follows: 0–3 min (20% B), 3–8 min (30% B), 8–18 min (30% B), 18-19 min (50% B), 19–40 min (50% B), and 40-41 min (90% B), at 1 mL/min flow velocity and 330 nm wavelength. 5 *μ*L of sample was injected into the column with an autosampler. Quantitative content of decursin (#KP013, NPC Biotech, Sejong, Korea) was analyzed via a photodiode array detector (996, Waters, MA, USA) interfaced to the separations module (Figure 1).

### 2.4. Induction of Colitis

After adaptation, mice were randomly divided into the following 4 groups (4–6 mice/group): normal control, colitic control, AAK 100, and AAK 500. Colitis was induced with DSS as previously described [28]. Briefly, 36–50 kDa DSS (#0216011080, MP Biomedicals, CA, USA) was dissolved in sterile tap water at a final concentration of 4% (wt/vol) and presented to mice as drinking water for 7 consecutive days. Normal controls were administered sterile tap water by oral gavage once per day, with free access to drinking water; colitic controls were also administered sterile tap water by oral gavage once per day, but with free access to DSS-treated drinking water; AAK 100 and AAK 500 mice were, respectively, administered 100 and 500 mg/kg of body wt AAK by oral gavage once per day, with free access to DSS-treated drinking water. The doses of AAK were chosen on the evidence of previously-demonstrated protective effects of* Angelica* root in multiple inflammatory disease models [22, 29–31]. Total gavage volume (200 *μ*L) was identical for each group.

### 2.5. Evaluation of Disease Activity

Mice were monitored daily for changes in body weight, stool consistency, and stool blood. Body weight on any particular day was calculated as the relative percentage weight to that of day 1. Stool consistency was evaluated macroscopically using the following 4-point scale: 0, formed and hard; 1, formed but soft; 2, loose stool; 3, watery. Traces of occult blood in feces were assessed using Hemoccult SENSA® single slides (#38078, Beckman Coulter, CA, USA) in the following 4-point scale: 0, hemoccult negative (−); 1, hemoccult positive (+); 2, hemoccult positive (++); 3, gross bleeding on the anus site. After sacrifice, the colon was removed and measured in length for evaluation of shortening.

### 2.6. Termination of Experiment and Sampling

After 6 h receiving the last gavage, mice were anaesthetized with avertin. Upon dissection, whole blood was collected by cardiac puncture and immediately centrifuged to isolate serum. After euthanization, spleen samples were removed and measured in weight. The full colon was also removed and measured in length, from the cecocolic junction to the proximal rectum. Following extraction of the cecum, fecal matter was removed. The colon was then washed in phosphate buffered saline (PBS), and the inflamed distal region was cut and fixed in 4% formaldehyde for histological examination. The remaining mid-proximal portion was dissected into fragments and stored at −80°C for further analysis.

### 2.7. Histopathological Evaluation

Fixed distal colon tissue samples were embedded in paraffin using a tissue processor (TP1020, Leica Biosystems, Nussloch, Germany). Paraffin blocks and 4 *μ*m section slides were made via conventional methods. Upon hematoxylin and eosin (H&E) staining, representative regions of the longitudinal and transverse sections were viewed using light microscopy at 100x/400x magnification, respectively. Each H&E stained sample was scored by the use of parameters previously described [32], with modifications. Briefly, a total score ranging from 0 (healthy) to 12 (extensive injury) was determined by the sum of respective scores for the following parameters: (1) epithelial erosion, (2) goblet cell depletion, (3) inflammatory cell infiltration, and (4) crypt deformation. Scoring of each parameter was defined as follows: 0, none; 1, mild; 2, moderate; 3, severe.

### 2.8. RNA Extraction and Real-Time PCR

Colon tissue samples were thawed and total RNA was extracted using the R&A-BLUE*™* kit (#17501, iNtRON Biotech., Seongnam, Korea) according to the manufacturer's protocol. Eluted RNA samples were quantified with the NanoDrop*™* spectrophotometer (Thermo Scientific, DE, USA) at 260 nm absorbance, and 2 *μ*g of RNA was subsequently reverse-transcribed using the PrimeScript*™* cDNA synthesis kit (#6110A, Takara, Shiga, Japan) according to the manufacturer's protocol. Real-time quantitative PCR was carried out on reaction tubes (#4358293, Applied Biosystems, CA, USA) and caps (#4323032, Applied Biosystems, CA, USA) in a StepOne*™* real-time PCR system (Applied Biosystems, CA, USA) with 100 ng of cDNA, the SensiFAST*™* SYBR Hi-ROX kit (#BIO-92005, Bioline, London, UK), and previously described primers for the targeted genes [33, 34]. Relative gene expression was calculated via the comparative Ct (2^−ΔΔCt^) method, and mouse glyceraldehyde 3-phosphate-dehydrogenase (GAPDH) was used as an endogenous control for normalization.

### 2.9. Enzyme-Linked Immunosorbent Assay (ELISA)

Upon whole blood collection via cardiac puncture, the serum was isolated by centrifugation at 1,500 rpm for 20 min at 4°C. Determination of serum IgE levels was performed using the BD OptEIA*™* mouse IgE ELISA set (#555248, BD Biosciences, CA, USA), according to the manufacturer's protocol. After the final step, plate was read with a microplate reader (VersaMax, Molecular Devices, CA, USA) at 450 nm absorbance.

### 2.10. Statistical Analysis

All statistical analyses were performed using the GraphPad Prism 5 (GraphPad Software Inc., CA, USA) software. Statistical significance for differences between curves was tested using two-way analysis of variance (ANOVA), followed by Bonferroni correction. Statistical significance for differences between means was tested using one-way ANOVA, followed by Newman-Keuls multiple comparison test. Data are presented as the mean ± SD, and *p* values of < 0.05, < 0.01, and < 0.001 were considered statistically significant.

## 3. Results

### 3.1. Oral AAK Ameliorates Colitis-Induced Anorexia and Weight Loss

**Figure 2:**
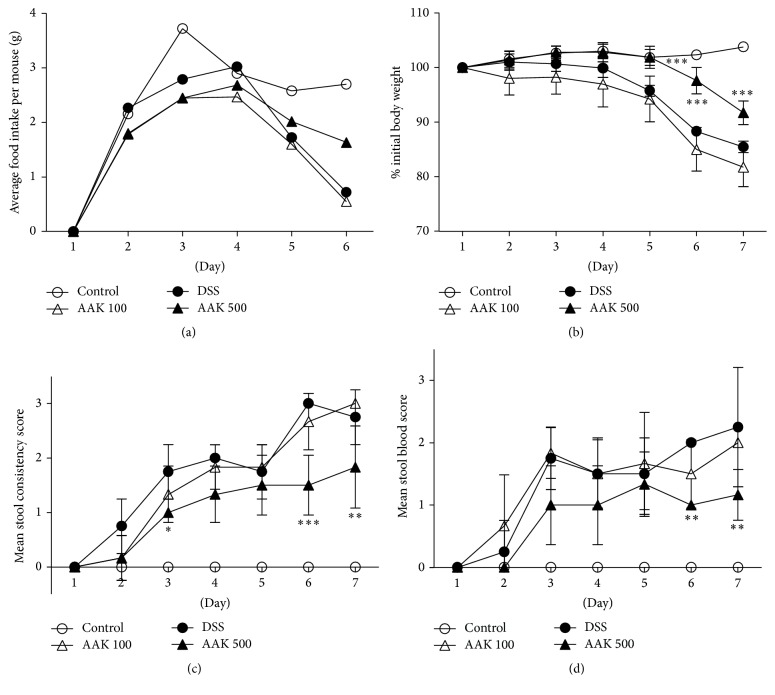
Effects of AAK extract on the symptoms of DSS-induced colitis. (a) Daily changes of food intake. Average food intake per mouse was calculated by dividing total consumption of chow on a specific day with the number of mice per cage. (b) Daily changes of body weight. Body weight was calculated by dividing weight on a specific day with the initial weight of each mouse. Stool samples were monitored daily for scoring of (c) consistency and (d) occult blood. Data represent the percentage or mean ± SD (*n* = 4–6 per group). ^*∗*^ *p* < 0.05; ^*∗∗*^ *p* < 0.01; ^*∗∗∗*^ *p* < 0.001 versus DSS.

Upon receiving DSS, mice developed acute colitis, which was evidenced by a reduction in average food intake and body weight. Oral administration of AAK (500 mg/kg/day) resulted in a higher daily average food intake compared to colitic controls with vehicle treatment (Figure 2(a)). Accordingly, significant reduction in body weight loss was observed in mice with AAK (500 mg/kg/day) administration, on days 5, 6, and 7 (Figure 2(b)). No significant differences between low-dose (100 mg/kg/day) AAK-treated mice and colitic controls were observed, in both average food intake and body weight.

### 3.2. Oral AAK Inhibits Development of Diarrhea and Stool Blood

Changes in stool consistency and occult blood were observed in mice upon exposure to DSS. As early as day 2, mice receiving DSS developed soft stools with mild traces of diarrhea and tested positive in the guaiac test. The severity of manifestations progressively intensified towards termination of experiment, where mice exhibited watery feces and gross bleeding on the anus site. In line with the effects on food intake and body weight loss, oral administration of AAK (500 mg/kg/day) resulted in a significant suppression of diarrhea events on days 3, 6, and 7 (Figure 2(c)). Oral AAK (500 mg/kg/day) also significantly protected against presence of occult blood on days 6 and 7 (Figure 2(d)). Again, no significant differences between low-dose (100 mg/kg/day) AAK-treated mice and colitic controls were observed, in the scores of both stool consistency and occult blood.

### 3.3. Oral AAK Ameliorates Colitis-Induced Colon Shortening and Splenomegaly

**Figure 3:**
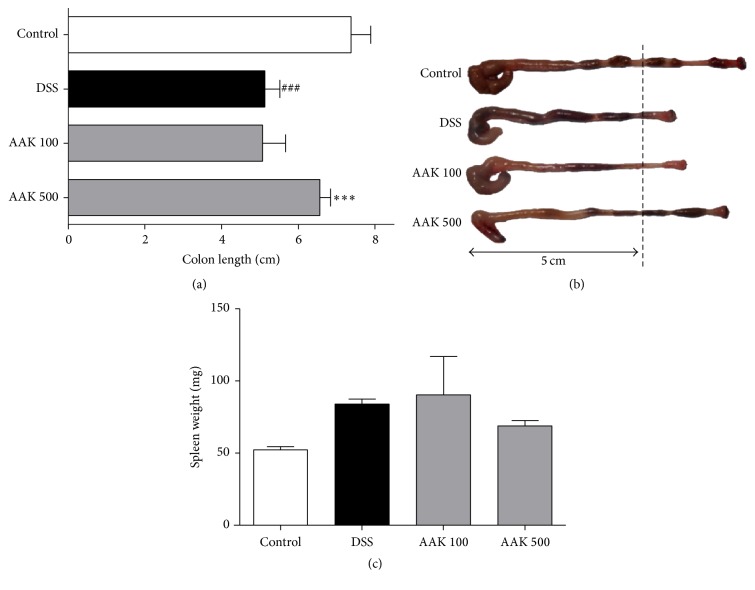
Effects of AAK extract on the colon length and spleen weight of DSS-induced colitis. Mice colon and spleen samples were collected on the day of sacrifice. (a) Colon length of each group. (b) Macroscopic images of the colon. (c) Spleen weight of each group. Data represent the mean ± SD (*n* = 4–6 per group). ^###^ *p* < 0.001 versus control; ^*∗∗∗*^ *p* < 0.001 versus DSS.

Immediately after the termination of experiment, colon and spleen samples were analyzed for their length and weight, respectively. Colitic controls with DSS had a significantly shortened colon length (5.1 ± 0.4 cm), compared to normal controls with vehicle treatment (7.3 ± 0.5 cm). Mice with oral AAK (500 mg/kg/day) administration had a significantly well-retained colon length (6.6 ± 0.3 cm) compared to colitic controls. As for the low-dose (100 mg/kg/day) AAK group, average colon length (5.1 ± 0.6 cm) was analogous to that of colitic controls (Figures 3(a) and 3(b)). In the case of spleen weight, oral AAK (500 mg/kg/day) administration showed a trend of reduction (68.9 ± 3.8 mg), although it did not reach statistical significance compared to colitic controls (84.1 ± 3.3 mg) (Figure 3(c)).

### 3.4. Oral AAK Protects against Microscopic Colon Damage

**Figure 4:**
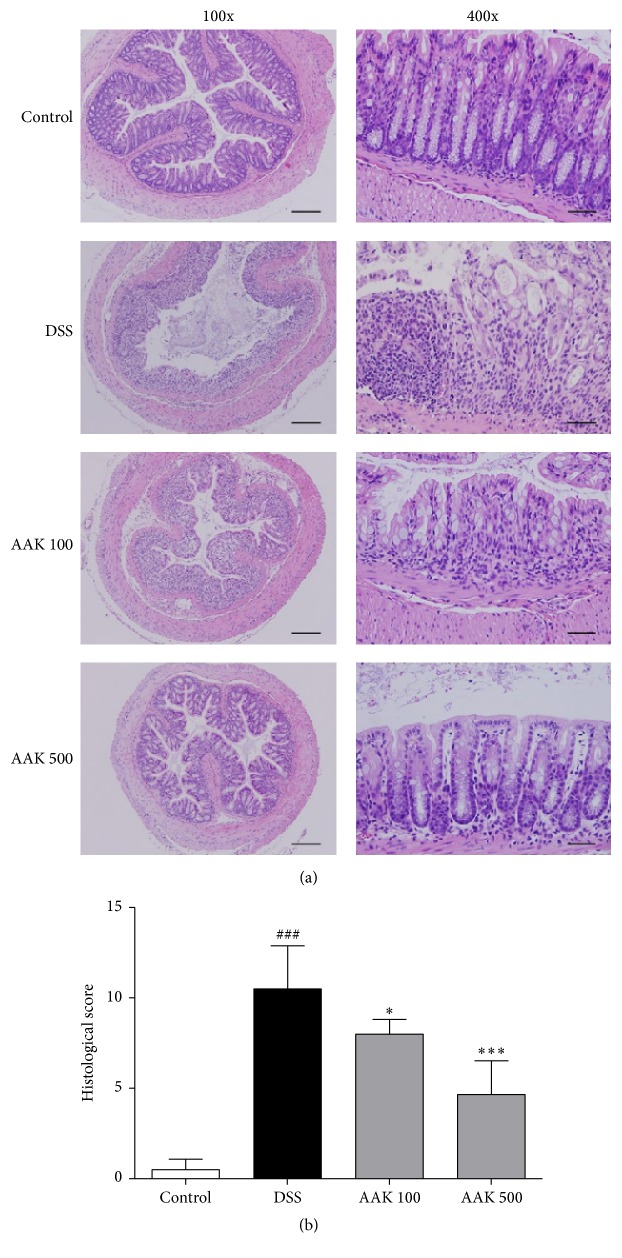
Effects of AAK extract on the colonic histological property of DSS-induced colitis. (a) Representative microscopic images of hematoxylin and eosin (H&E) stained colon sections in each group. Scale bars indicate 400 *μ*m for transverse sections (100x) and 100 *μ*m for longitudinal sections (400x). (b) Cumulative histology scores for each group. Data represent the mean ± SD (*n* = 4–6 per group). ^###^ *p* < 0.001 versus control; ^*∗*^ *p* < 0.05 and ^*∗∗∗*^ *p* < 0.001 versus DSS.

Histological assessment of distal colon samples revealed that DSS-treated mice exhibited loss of surface epithelium, villous blunting, crypt distortion or loss, goblet cell loss, and inflammatory cell infiltration. While normal controls showed mild or no signs of histological damage (score 0.5 ± 0.6), colitic controls had marked severe morphological changes throughout the epithelium and mucosa, with a dense transmural inflammatory cell infiltration (score 10.5 ± 2.4). The colons of oral AAK-treated mice showed substantial protection against such damage. At high-dose (500 mg/kg/day) AAK administration, mucosal architectures and goblet cells were relatively well-preserved, with a sparse distribution of inflammatory cells in the lamina propria (score 4.7 ± 1.9). Low-dose (100 mg/kg/day) administration of AAK did exhibit signs of amelioration, but not to the same extent (score 8 ± 0.8) (Figure 4).

### 3.5. Oral AAK Suppresses mRNA Expression of Inflammatory Mediators

**Figure 5:**
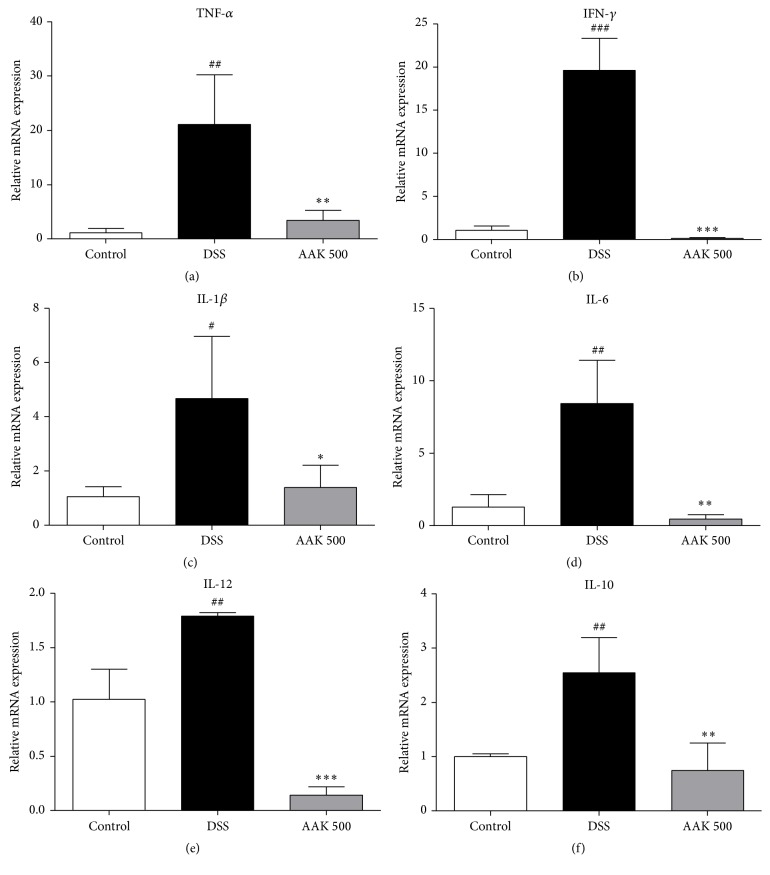
Effects of AAK extract on the gene expression levels of inflammatory mediators in DSS-induced colitis. (a–f) mRNA expression of TNF-*α*, IFN-*γ*, IL-1*β*, IL-6, IL-12, and IL-10 was measured by real-time PCR, and GAPDH was used as an endogenous control. Fold-changes are expressed as the mean ± SD (*n* = 4–6 per group). ^#^ *p* < 0.05, ^##^ *p* < 0.01, and ^###^ *p* < 0.001 versus control; ^*∗*^ *p* < 0.05, ^*∗∗*^ *p* < 0.01, and ^*∗∗∗*^ *p* < 0.001 versus DSS.

**Figure 6:**
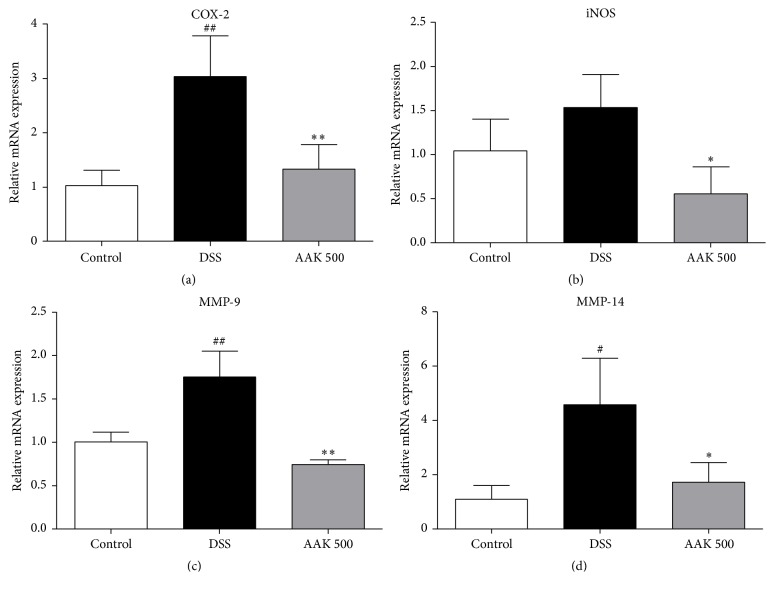
Effects of AAK extract on the gene expression levels of inducible enzymes and MMPs in DSS-induced colitis. (a–d) mRNA expression of COX-2, iNOS, MMP-9, and MMP-14 was measured by real-time PCR, and GAPDH was used as an endogenous control. Fold-changes are expressed as the mean ± SD (*n* = 4–6 per group). ^#^ *p* < 0.05 and ^##^ *p* < 0.01 versus control; ^*∗*^ *p* < 0.05 and ^*∗∗*^ *p* < 0.01 versus DSS.

**Figure 7:**
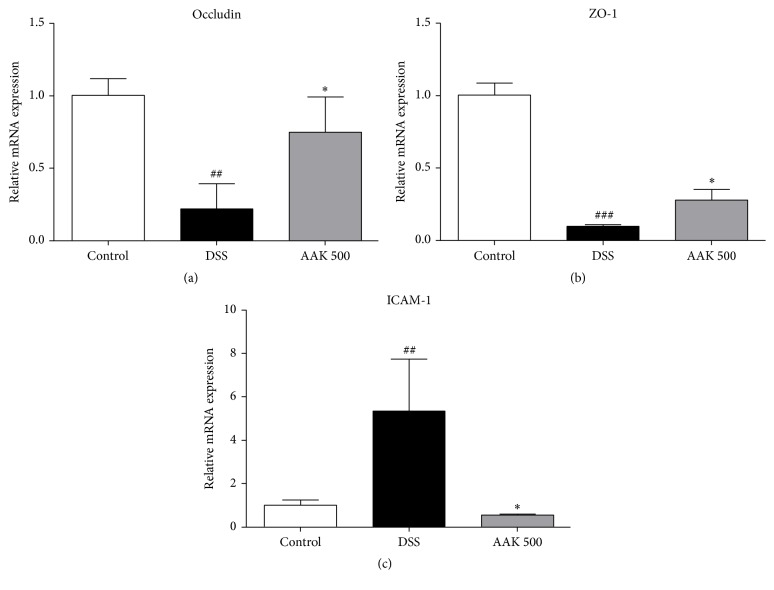
Effects of AAK extract on the gene expression levels of tight junction-related proteins in DSS-induced colitis. (a–c) mRNA expression of occludin, ZO-1, and ICAM-1 was measured by real-time PCR, and GAPDH was used as an endogenous control. Fold-changes are expressed as the mean ± SD (*n* = 4–6 per group). ^##^ *p* < 0.01 and ^###^ *p* < 0.001 versus control; ^*∗*^ *p* < 0.05 versus DSS.

To evaluate the mitigative effects of AAK on DSS-mediated immune response, expression of inflammatory cytokines, inducible enzymes, matrix metalloproteinases (MMPs), and tight junction-related proteins was measured at mRNA levels. Intestinal inflammation in colitic controls was characterized by a marked increase in pro- and anti-inflammatory cytokines TNF-*α*, IFN-*γ*, IL-1*β*, IL-6, IL-12, and IL-10, compared to normal controls. Administration of AAK (500 mg/kg/day) was able to significantly suppress the elevation of all these cytokines (Figure 5). Regarding the expression of inducible enzymes iNOS and COX-2, oral AAK (500 mg/kg/day) significantly suppressed both, which were elevated upon DSS administration. Similar patterns of amelioration were observed in the expression of MMP-9 and MMP-14 (Figure 6). While degraded epithelial integrity was evident in the marked decrease of occludin and ZO-1 expression upon DSS administration, oral AAK (500 mg/kg/day) was capable of restoring it. In the case of ICAM-1, an increase was present upon DSS administration, which was successfully reduced to the basal level in oral AAK (500 mg/kg/day)-treated mice (Figure 7).

### 3.6. Oral AAK Reduces Serum IgE Levels

**Figure 8:**
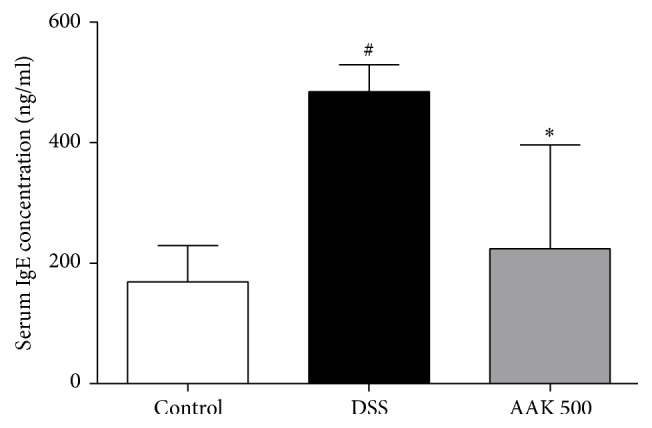
Effects of AAK extract on the concentrations of serum IgE in DSS-induced colitis. Total serum IgE levels were measured by ELISA. Values are expressed as the mean ± SD (*n* = 4–6 per group). ^#^ *p* < 0.05 versus control; ^*∗*^ *p* < 0.05 versus DSS.

In DSS-treated colitic controls, serum total IgE levels were upregulated by approximately twofold (484.4 ± 44.4 ng/mL), compared to that of normal controls (168.6 ± 60.2 ng/mL). In mice with oral AAK (500 mg/kg/day) administration, serum total IgE levels were significantly downregulated (224 ± 172.3 ng/mL) (Figure 8).

## 4. Discussion

Clinical response to standard therapy among UC patients is discrete, depending on the combination of factors that eventually led to the disease [5]. Despite recent advances in therapeutic regimen, a considerable portion of patients with UC still fail or lose response to conventional nonbiologic agents, which results in varied complications such as fibrostenosis that may require surgical intervention [6]. Approximately 11% of patients who use immunomodulators such as azathioprine or methotrexate discontinue administration due to allergic reactions, nausea, or pancreatitis. In the case of corticosteroids, 55% of patients end up in cessation of therapy as a result of recurring acne, facial swelling, and osteoporosis [35]. While biological agents (e.g., anti-TNF monoclonal antibodies) emerge as an alternative option, they have also been held by secondary responses that include the risk of hypersensitivity, immunogenicity, infection, and congestive heart failure [6]. Blockade of a single cytokine as such may give rise to an alternative compensatory pathway and thus is ineffective in many cases [36]. Herbal decoctions comprise a number of components that modulate the expression of multiple cytokines simultaneously [37] and therefore could prove as an effective complementary or alternative modality for UC patients. Nonadherence to treatment is yet another problem in the standard management of UC, which is caused by inconveniences following administration of large number of tablets, fear of side effects, or doubts in the need for medication during disease quiescence [38]. As phytotherapy is highly accepted by UC patients for its efficacy, low cost, and relative safety [39], it may also prove as a promising confrontation for such patient-derived nonadherence issues.

In this study, we demonstrated the effects of AAK extract in a DSS-induced acute colitis model. AAK belongs to the Umbelliferae family, along with* A. gigas* and* A. sinensis*. Roots of the* Angelica* plant are known to be rich in polysaccharides that exert immunomodulatory [21] and anti-inflammatory [24, 25] effects. The polysaccharides from* A. sinensis*, which is largely similar in composition to that of AAK, showed protective effects in TNBS and DNBS-induced rat models of UC [29, 40, 41]. While the root extracts from AAK have been reported to suppress inflammatory mediators in mouse macrophages [17], analogous effects on a murine UC model are not yet confirmed. Hence this study was aimed at investigating the effects of AAK extract on a DSS-induced model of UC. As previously reported, intestinal inflammation in the DSS-induced model of UC is primarily manifested as symptoms comparable to those of human-derived UC, such as anorexia, body weight loss, diarrhea, fecal occult blood, and colon shortening [9, 10, 42]. In patients with IBD, the most critical etiological factor for body weight loss is prolonged anorexia [43]. Decreased appetite in UC patients has been reported to be the result of increased serum leptin levels, which in turn sends a negative feedback signal to the hypothalamus [44, 45]. As for animal models, a comparable elevation in serum leptin, as well as the consequent loss of body weight, has been observed in the early stages of TNBS-induced intestinal inflammation in rats [46]. In line with these reports, the colitic group in our study exhibited a decrease in average food intake and body weight over the days of DSS administration. The ethanol extract of AAK was able to attenuate anorexia and subsequent weight loss, as evidenced by a relatively well-maintained food consumption and body weight at the terminal stage of experiment. Events of diarrhea and fecal occult blood were also significantly suppressed during the same period, both of which represent impaired epithelial barrier function and mucosal damage [47, 48]. For unknown reasons, the colonic longitudinal muscle undergoes hypertrophy and subsequent contraction during chronic UC. This contraction produces a diffuse or segmental narrowing of the lumen, which in turn causes shortening of the colon, namely, the “lead pipe sign” [49]. In this study, colonic length was significantly retained upon oral administration of AAK, compared to colitic controls. In animal models of UC, splenomegaly is validly indicative of intestinal inflammation, and mitigative effects of therapeutic substances have often been confirmed by the suppression of spleen enlargement [50, 51]. Mice with AAK showed a suppressive tendency in regard to spleen weight increase.

Cytokines control different aspects of the inflammatory response in UC. The imbalance between pro- and anti-inflammatory cytokines disrupts ease of inflammation and further leads to an elongation of the disease [36]. In line with such reports, cytokine-based therapies such as anti-TNF-*α* factors have recently been adopted in the UC-treatment algorithm [52]. The murine DSS-induced colitis model exhibits a distinct cytokine profile with elevated levels of TNF-*α*, IFN-*γ*, IL-1*β*, IL-6, and IL-12 [11]. While the models used in this study displayed similar patterns, our data suggest that oral administration of AAK significantly suppresses mRNA expression of such inflammatory cytokines in the inflamed colonic tissue. The proinflammatory cytokines TNF-*α* and IFN-*γ* play a pivotal role in the pathogenesis of UC, by altering the tight junction and inducing apoptosis of intestinal epithelial cells [53–55]. In Caco-2 cell monolayers, TNF-*α*-induced epithelial permeability was mediated by the expression of myosin-light chain kinase (MLCK) [56]. In the apical membrane of T84 cells, IFN-*γ* induced redistribution of occludin, claudin-1 and claudin-4, away from the tight junction domain [57]. The therapeutic potency of targeting these cytokines have been demonstrated in actual clinical trials with anti-TNF-*α* antibodies [52], as well as in* in vivo* studies where colitis showed attenuation upon DSS stimulation in IFN-*γ* ^−/−^ mice [58]. In the inflamed mucosa of IBD patients, key antigen-presenting cells (APCs) produce considerable amounts of proinflammatory cytokines such as IL-1*β*, IL-6, and IL-12 [59]. When compared to healthy subjects, patients with UC manifested a significant reduction in the ratio of IL-1 receptor antagonist to IL-1, which implies the augmented activation of IL-1 system in UC [60]. In a previous study using DSS-induced model of colitis, mice deficient in IL-1*β*-converting enzyme (caspase-1) exhibited attenuated signs of intestinal inflammation, suggesting the therapeutic relevance of IL-1 family blockade [61]. In experimental colitis, macrophages and CD4^+^ T cells of the lamina propria produce an increased amount of IL-6 [62, 63]. Upon binding with soluble IL-6R (sIL-6R), the IL-6-sIL-6R complex induces proinflammatory cytokine production in intestinal APCs and T cells, by binding to the gp130 surface molecule [36, 62]. The therapeutic potentials of IL-6 blockade have been confirmed in a previous study by Yamamoto et al., where administration of anti-IL-6R monoclonal antibody to a murine model of colitis resulted in reduced disease activity and suppression of proinflammatory cytokines [64]. IL-12 is a heterodimeric cytokine produced by monocytes and macrophages in response to bacteria or their products [65, 66]. By inducing IFN-*γ* synthesis and Th1 cell differentiation, IL-12 contributes to the breakdown of tolerance against luminal antigens in human and murine colitis [67]. Since IFN-*γ* upregulates IL-12 production in macrophages, the established loop between IL-12 and IFN-*γ* further contributes to the perpetuation of intestinal inflammation [68]. As neutralizing antibodies to IL-12 have shown suppressive effects on chronic intestinal inflammation, targeting IL-12 in UC may also prove as an effective means of treatment [69, 70]. The anti-inflammatory cytokine IL-10 is known to exhibit immunoregulatory properties by blocking the production of proinflammatory cytokines in monocytes and macrophages [71]. Restoration of endogenous IL-10, as well as administration of its exogenous components, has been proposed as a possible treatment for IBD [72]. However, such is not the case in this study, as the level of IL-10 expression was upregulated upon DSS administration and downregulated by AAK treatment. Similar patterns have been reported in previous studies with murine [34, 73] and porcine [74] models of intestinal inflammation. One possible explanation may be the rapid return to a homeostatic cytokine milieu upon treatment, as the endogenous production of IL-10 may have been insufficient to downregulate proinflammatory cytokines, and thus local inflammation [71, 74]. In further studies, the expression of anti-inflammatory cytokines besides IL-10, such as IL-4, should be examined to confirm the contribution of other endogenous factors. Taken together, the suppressive effects of AAK on inflammatory mediators of the above imply its possibility as a potent complementary and alternative therapy against UC.

Oral administration of AAK also significantly suppressed the mRNA expression of inducible enzymes and MMPs in the inflamed tissues of DSS-induced colitic mice. Elevated expressions of iNOS and COX-2 in the morbid colonic mucosa indicate production of nitric oxide (NO) and prostaglandin E2 (PGE_2_), two of the bioactive agents involved in colonic injury [75]. Recent studies have suggested a synergetic action of both enzymes, as inhibition of NO synthesis resulted in a reduced secretion of PGE_2_. Combined blockage of iNOS and COX-2 reportedly had a cytoprotective effect under UC conditions [76], and analogous data were drawn in the present study using AAK. MMPs are endopeptidases that degrade the extracellular matrix (ECM) components during active flares of UC. MMPs are capable of shedding molecules such as TNF-*α*, IL-1, and IL-6 from macrophage surfaces [77]. In this study, expression of MMP-9 and MMP-14 were downregulated upon AAK treatment. Mucosal recovery of AAK-treated mice was evident in our histological data, which was also confirmed by the restored mRNA expression of tight junction proteins ZO-1 and occludin. These linker and transmembrane proteins associate with each other to maintain epithelial integrity [78]. Lastly, our study demonstrated the suppressive effects of AAK on total serum IgE concentrations. Patients with UC have reported suffering from allergic diseases such as asthma, allergic rhinitis, or eczema, twice as much as matched healthy controls [79]. In another study by D'Arienzo et al., total serum IgE levels were accordingly higher in UC patients, compared to healthy controls [80]. Our results indicate that oral AAK extract may also have beneficial effects in treating the secondary atopic manifestations following UC.

Taken together, this study suggests that oral administration of AAK exerts a protective effect against the pathogenesis of acute colitis, by improving major symptoms such as anorexia, weight loss, diarrhea, occult blood, and colon shortening. Furthermore, we demonstrated that such attenuative effects are partially mediated by the suppression of multiple inflammatory markers, including cytokines, inducible enzymes, MMPs, and tight junction-related proteins. These findings provide the possibilities for a more sustainable approach towards UC, as conventional therapeutics with a single-cytokine target are likely to have limitations due to development of compensatory pathological pathways [36]. In the present study, AAK mostly regulated APC-derived cytokines. However, recent studies have suggested that nonimmune cells, such as epithelial cells or stromal fibroblasts, may also produce inflammatory cytokines in IBD [36]. In view of such findings, we may hypothesize that AAK elicits an innate immune cell-mediated response, while targeting the epithelium itself at the same time. However, as our data do not confirm a predominant activation of either mechanism, further* in vivo* and* in vitro *experiments are required to define the exact mode-of-action responsible for the protective activities of AAK.
